# Fractional Bandwidth up to 24% and Spurious Free SAW Filters on Bulk 15°YX-LiNbO_3_ Substrates Using Thickness-Modulated IDT Structures

**DOI:** 10.3390/mi13030439

**Published:** 2022-03-14

**Authors:** Zengtian Lu, Sulei Fu, Zhibin Xu, Weibiao Wang, Qiaozhen Zhang, Jianrun Zhang, Hui Zhang

**Affiliations:** 1 School of Mechanical Engineering, Southeast University, Nanjing 211189, China; luzengtian@wxsde.cn (Z.L.); ajitamjaya_hri@163.com (Z.X.); zhangjr@seu.edu.cn (J.Z.); 2 Key Laboratory of Advanced Materials (MOE), School of Materials Science and Engineering, Tsinghua University, Beijing 100084, China; 3 Shoulder Electronics Limited, Wuxi 214124, China; wbwang1970@163.com; 4 College of Information, Mechanical and Electrical Engineering, Shanghai Normal University, Shanghai 200234, China; zhangqz@shnu.edu.cn

**Keywords:** surface acoustic wave, filter, wideband, spurious mode, LiNbO_3_

## Abstract

To cope with ubiquitous wireless connectivity and the increased and faster data delivery in 5G communication, surface acoustic wave (SAW) filters are progressively requiring wider bandwidths. Conventional bulk 15°YX-LiNbO_3_ substrates with a large coupling coefficient (*K*^2^) are attractive for the low-cost mass production of wideband SAW filters, but these generally suffer from spurious responses, limiting their practical application. In this work, a novel and simple SAW configuration is proposed that uses thickness-modulated interdigital transducer (IDT) structures to overcome the limitations set by spurious responses. Different from the conventional design where the thicknesses of the IDT electrodes in the series and parallel resonators generally kept the same, the proposed configuration adopts IDT electrodes of different thicknesses in the series and shunt resonators to suppress or remove unwanted spurious Rayleigh modes from the filter passband. Two different ultra-wideband SAW filter designs employing thickness-modulated IDTs were designed and fabricated to validate the effective suppression of spurious modes. The SAW filters experimentally featured spurious-free responses in the passband as well as a large 3 dB fractional bandwidth (FBW) in the 18.0% and 24.1% ranges and low insertion losses below 1 dB. This work can significantly broaden the range of applications for SAW devices and can open a pathway to commercialize ultra-wideband SAW filters in 5G communication systems.

## 1. Introduction

Surface acoustic wave (SAW) filters exhibit excellent frequency band selectivity and are small, serving as an irreplaceable component in the front-end radio frequencies (RF) required for modern communication [1,2,3,4,5]. To fulfill the explosive growth of data transmission demands and ubiquitous wireless connectivity in 5G communications, significantly wider bandwidths (BW) are required for 5G compared to previous generations of communication technologies [6,7,8,9]. For example, the filters for new 5G bands can currently deliver a fractional bandwidth (FBW) of more than 13% (compared to 3–5% for 4G) [9,10]. Consequently, the demand for SAW filters with a higher BW is continuously pursued and never seems to subside.

While a larger BW presents a big challenge for the performance levels of commercial off-the-shelf SAW filters, their intrinsic restrictions, i.e., the low electromechanical coupling coefficient (*K*^2^), lead to a typically limited FBW of up to 3% [11]. Several approaches have been proposed and carried out to overcome this limitation and to improve the BW of filters, such as by adopting passive electromagnetic component-aided technologies, utilizing piezoelectric films on insulator layered structures, or using plate acoustic wave modes [12,13,14,15,16]. For example, we recently reported on wideband SAW filters with a large 3-dB FBW of over 20% on LiNbO_3_ that had been film-bonded on a handling substrate [17,18]. However, the fabrication processes of LiNbO_3_ film-bonded substrates are complex and require the precise control of each film’s thickness. Moreover, the aforementioned improvements are also acquired at the expense of additional insertion losses, filter volumes, or costs.

Alternatively, shear-horizontal (SH) type SAWs on the well-known rotated bulk YX cuts of lithium niobate (YX-LiNbO_3_) substrates are promising for enabling the low-cost mass production of wideband SAW filters due to their ultra-large *K*^2^ of up to 30% and commercial availability from wafer vendors [19]. K. Hashimoto and coworkers have reported on SAW devices on a Cu-grating/15°YX-LiNbO_3_ structure [20,21,22], setting a milestone for fabricating ultra-wideband SAW filters that are low-cost and that have a simple fabrication process. Nevertheless, the SAW filters demonstrated many spurious resonances in a measured response, making them somewhat tricky for practical application.

Efforts have been made to suppress those spurious modes on the LiNbO_3_ substrate by employing many, methods such as width/length-weighted dummy IDT electrodes, apodized IDT electrodes, or modified rhombic weighted IDT electrodes [23,24,25]. These works mainly focus on suppressing transverse modes. Indeed, the transverse modes in SAW filters can be effectively blurred out using weighted IDT electrodes. However, the problem of another type of spurious response, referred to as the Rayleigh mode, which inevitably exists in SAW filters, has not been solved effectively in previously reported works, causing serious ripples or in-band fluctuations in the filter passband. Although T. Omori et al. reported the suppression of the Rayleigh mode by coating the devices with a viscous film, it could not be suppressed entirely and still caused ripples in the filter passband [22]. As a result, it represents a long-standing bottleneck for further advancing spurious wideband SAW filters on YX-LiNbO_3_ substrates into real-life applications.

To address this issue, this work focuses on managing spurious Rayleigh modes on 15°YX-LiNbO_3_ substrates. To this end, first, we started with a theoretical analysis to explore the influence of Cu electrode thickness on the spurious responses caused by the Rayleigh mode. Then, we analyzed why spurious responses always exist in conventional SAW filters. Next, two SAW configurations using double thickness-modulated IDTs in series and shunt resonators to suppress unwanted spurious modes in the filter passband were proposed and analyzed. Ladder-type SAW filters with a large 3 dB FBW in the 18.0% and 24.1% ranges and employing thickness-modulated IDTs were designed and fabricated on 15°YX-LiNbO_3_ substrates to validate our analysis and modeling. It was confirmed that the filters were measured with the spurious-free response. Compared to previous work based on LiNbO_3_ film-bonded substrates [17,18], the device performance, which includes the frequency, FBW, and passband flatness, in this work are simultaneously improved. Moreover, the complexity of the manufacturing processes and the preparation costs are greatly reduced. Our work shows the feasibility of ultra-wideband and spurious-free filters on conventional bulk LiNbO_3_ substrates for bands with a higher BW in front-end RF via current SAW technology.

## 2. Materials and Methods

Quasi-3D FEM simulations of the acoustic propagation characteristics of the Rayleigh and SH modes on the 15°YX-LiNbO_3_ at different normalized electrode thicknesses *h*_Cu_*/λ* were carried using the structural mechanics module in COMSOL software [26]. In the FEM model, Cu is defined as the IDT electrode material, and the wavelength λ and metallization ratio of the SAW resonators are 2 μm and 0.5, respectively. In the *x* direction, the number of IDT fingers and the number of reflector fingers are set to 89 and 20, which is more favorable for studying the influences of the actual device configurations. Compared to the conventional calculation method (both *x* and *y* are set as periodic), this model, which has been configured with a finite length in both the *x* and *z* directions, should call for more computing resources; thus, perfect matching layers with a width of 1 *λ* were set on the sidewalls to reduce the size of the model and to suppress the boundary reflections. The mesh type used had free tetrahedral elements, and the maximum element size was set as *λ*/10. The resonance frequency (*f*_r_) and the anti-resonance frequency (*f*_a_) of SAW devices can be extracted from the calculated admittance responses using frequency domain analysis [27]. Additionally, the SAW characteristics of devices, such as the resonance velocity (*V*_r_), the anti-resonance velocity *(V*_a_), and electromechanical coupling coefficient *K*^2^ can be determined according to the methods in [28,29].

The SAW filters were fabricated using the conventional photolithography and lift-off processes. The IDT electrodes were deposited via e-beam evaporation. Since SAW filters are packaged in actual applications to protect the die surface from damage and contamination, the influence of the package is also considered during filter design The SAW filters in this work were packaged using a standard surface-mounted device or using chip-scale packages. The RF performance of the SAW filters was measured using an Agilent E5071C vector network analyser (VNA). For resonator measurements, SAW resonators were tested on a Cascade M150 probe station with a GSG probe, and a standard impedance substrate was used to calibrate the RF probes for short, open, and load measurements. The calibration transferred the measurement plane from the VNA to the tip of the RF probe. For the filter measurements, Keysight N4431D electronic calibration (ECal) was used to calibrate the VNA. 

## 3. Results and Discussion

### 3.1. Theoretical SAW Characteristics and Traditional Filter on 15°YX-LiNbO_3_

**Figure 1:**
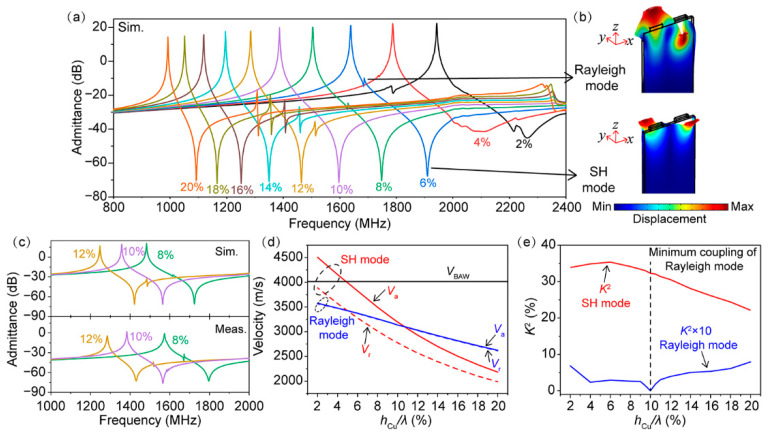
(**a**) The simulated admittance curves at different normalized thicknesses *h*_Cu_/λ on 15°YX-LiNbO_3_ substrate; (**b**) displacement field of Rayleigh and SH modes; (**c**) the simulated and measured admittance curves at normalized thicknesses *h*_Cu_/λ of 8%, 10% and 12%; (**d**,**e**) velocities and electromechanical coupling coefficients of two modes extracted from the simulated admittance curves.

Figure 1a illustrates the simulated admittance responses of the SAW resonators at different normalized electrode thicknesses *h*_Cu_/*λ* from 2% to 20%. Two acoustic resonances were present, which corresponded to the SH mode and Rayleigh mode as determined through the mode shape analysis shown Figure 1b. Rayleigh mode interacted with the SH mode and perturbed the frequency response of the desired SH mode. When the thickness of the Cu electrode was below 4%, the anti-resonance characteristic of the SAW device was weak, indicating the leaky characteristic of the SH mode. As the IDT electrode thickness increased, a normal admittance ratio was achieved. As the electrode thickness increased, Rayleigh mode first decreased to near zero at *h*_Cu_/*λ* = 10% and then increased slightly, thus resulting in small tips above the anti-resonance frequency of SH mode. To verify the FEM simulations, resonators with λ = 2 μm were fabricated and tested. To obtain the intrinsic SAW properties, the parasitic effect of the resonator was de-embedded by subtracting the measurement results of the open and short structures. Figure 1c displays the simulated and measured admittance curves of the resonators with *h*_Cu_/*λ* of 8%, 10%, and 12%. Note that compared to these strong spurious modes at 8% and 12%, the measured resonators only show a neglectful tiny spurious mode above the anti-resonance frequency. Moreover, the measured admittance characteristics were consistent with the calculated results. The small deviation between the calculated and measurement results can be attributed to several aspects, such as the differences in the material parameters between the calculated and the experimental values, the inaccuracy of the experimental IDT thickness and shape, material defects, and finite length in the *y* direction. The simulated and measured results both indicate that the minimum coupling was nearly zero for Rayleigh mode at *h*_Cu_/*λ* about 10%.

Furthermore, based on the calculated admittance curves, the dispersion of *V*_r_, *V*_a_, and *K*^2^ for the two modes were extracted, as shown in Figure 1d,e. It can be seen that when *h*_Cu_/*λ* was larger than 5%, enough of the heavy electrodes ensured that *V*_r_ and *V*_a_ of the SH mode were lower than the slow bulk acoustic wave velocity (~4029 m/s) of LiNbO_3_. Thus, the SH mode became non-leaky, agreeing with previous work [20]. Compared to the SH mode, Rayleigh mode presented a much smaller *K*^2^ that was below 1% in the *h*_Cu_/*λ* interval of 2–20%. Minimum coupling was nearly zero for Rayleigh mode at an *h*_Cu_/*λ* of about 10%, with the SH mode still maintaining a high *K*^2^ of over 30%. As a spurious response, the coupling of the undesired Rayleigh mode was expected to be as small as possible. Therefore, a *h*_Cu_/*λ* of around 10% was considered to be the optimal thickness for developing a high-performance wideband SAW filter.

**Figure 2:**
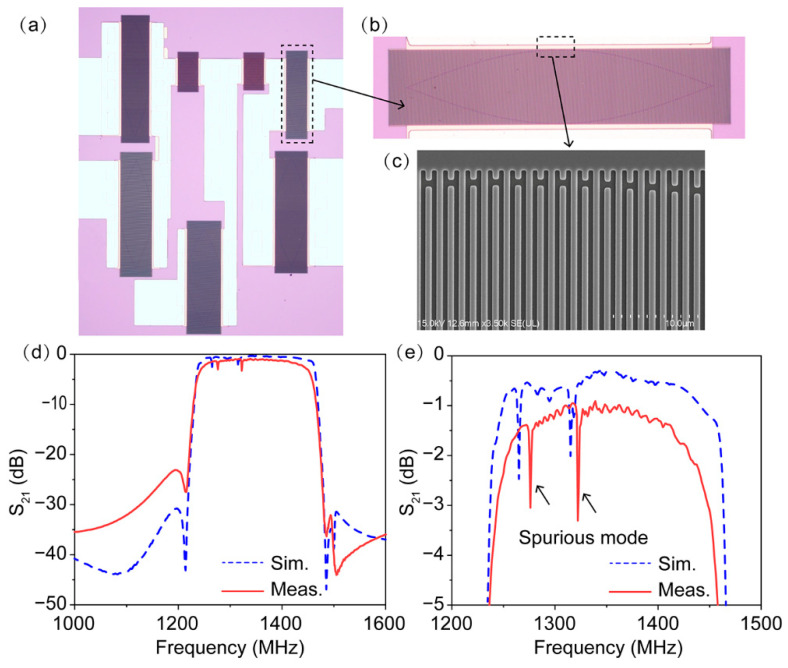
(**a**) Optical microscope image of the fabricated filter using the conventional design; (**b**) zoomed-in optical microscope image; (**c**) SEM image of the IDT fingers; (**d**) simulated and measured frequency responses of the filter; (**e**) zoomed-in simulated and measured frequency responses of the filter.

Based on the aforementioned results, the experimental ladder-type SAW filters were designed and fabricated on the 15°YX-LiNbO_3_ substrate using the conventional design. In other words, the IDT electrodes of the SAW filters were via one-step photolithography. Figure 2a shows an optical image of the fabricated filter, which consists of four series and three parallel resonators. Here, it should be mentioned that all of the SAW filters in our work adopted a weighted IDT design to subdue spurious transverse modes, as shown in Figure 2b. The scanning electron microscope (SEM) image shown in Figure 2c indicates that the IDTs have high regularity and well-proportioned patterns. The wavelengths λ of the resonators ranged from 1.60 μm to 2.60 μm, and the thickness of IDT electrodes was chosen to be 210 nm. Figure 2d,e display the simulated and measured frequency responses of the filter. The measured performance agrees well with the simulated frequency response. It can be clearly seen that the measured filter had a center frequency (*f*_c_) of 1347 MHz, a minimum insertion loss (IL_min_) of 1.0 dB, and a large 3-dB FBW of 15.9% (BW = 214 MHz). However, both the simulated and measured frequency responses indicate that obvious in-pass ripples resulting from Rayleigh modes existed in the passband using the conventional design, leading to large in-band fluctuations of over 2 dB and deteriorated performance. Similar results have also been reported in previous works [21,22]. Because spurious Rayleigh modes unavoidably disturb the frequency response of the filters, the development of a spurious-free wideband SAW filter on a 15°YX-LiNbO_3_ substrate has seemed impossible to achieve and accomplish until now.

### 3.2. Conventional Filter Configuration and Thickness-Modulated IDT Filter Configuration

**Figure 3:**
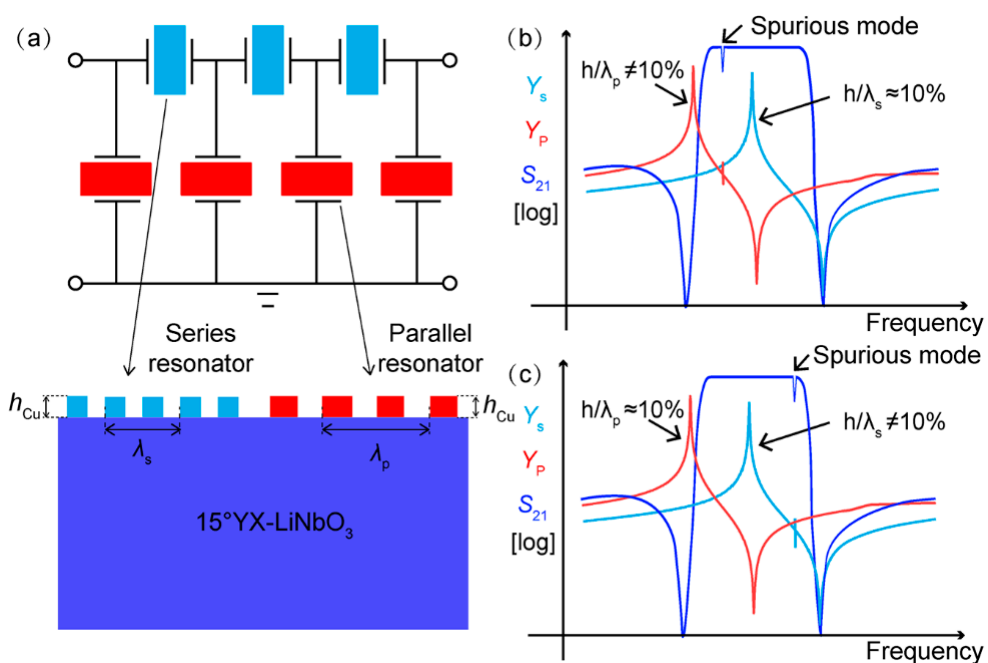
(**a**) The basic structure of a conventional ladder-type SAW filter. (**b**,**c**) Schematic diagram of the existence of spurious modes in the conventional filter.

To reveal the reason for the existence of spurious mode in the filters, we need insight into the configuration of the traditional filter. Figure 3a shows a typical configuration for a conventional adder-type filter that employs resonators in both series and parallel branches. In order to obtain a bandpass response, the resonant frequency of the series resonator is usually designed to be nearly coincident with the anti-resonance frequency of the parallel resonator. Therefore, the wavelengths of the series and parallel resonators (*λ*_s_ and *λ*_p_) in their constructed filter are designed to be different to attain the frequency offset [12]. For the traditional design, the IDT electrodes are made by means of one-step photolithography. In other words, the thicknesses of the electrodes (*h*_Cu_) in the series and parallel resonators are the same. As a result, the normalized electrode thicknesses for the series and parallel resonators (*h*_Cu_/*λ*_s_ and *h*_Cu_/*λ*_p_) are different. Thus, the optimal normalized thickness of around 10% cannot be obtained simultaneously in the resonators comprising the SAW filters. From the results in Figure 1, it is known that Rayleigh mode is sensitive to deviations in electrode thickness. As plotted in Figure 3b, when the optimal *h*_Cu_/*λ*_s_ ≈ 10% is set for the series resonators, the *h*_Cu_/*λ*_p_ of the parallel resonators is not 10%. As a result, spurious modes will occur in the admittance curve of the parallel resonators, causing ripples in the filter passband. Similarly, when the optimal *h*_Cu_/*λ*_p_ ≈ 10% is chosen for the parallel resonators, *h*_Cu_/*λ*_s_ of series resonators is not 10%. Spurious modes also appear in the filter passband, as shown in Figure 3c. That is why the wideband SAW filters on the 15°YX-LiNbO_3_ substrate have so far been able to confront the spurious responses in the passband. Therefore, a more elaborate and optimized management of spurious Rayleigh modes is required to ensure that the filters have a spurious-free passband.

**Figure 4:**
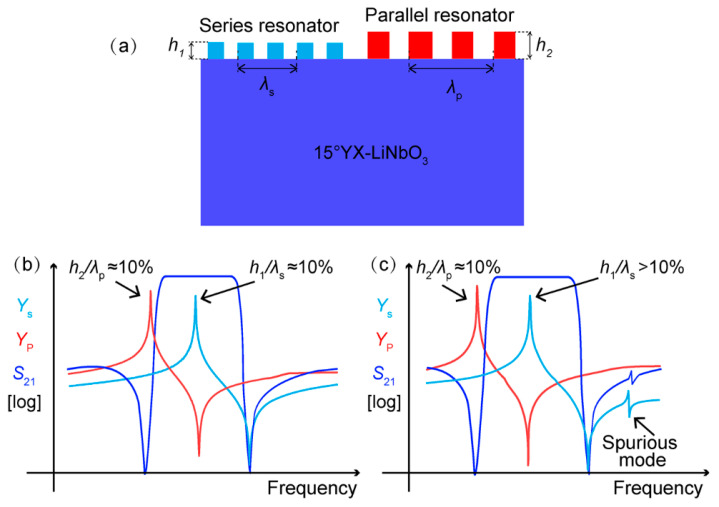
(**a**) Design of thickness-modulated IDT structures in this work. (**b**,**c**) Schematic diagrams of two thickness-modulated IDT designs.

To circumvent the limitations set by different *h*_Cu_/*λ* in the series and parallel resonators in a traditional SAW design, a thickness-modulated structure was proposed by applying different IDT electrode thicknesses in the series and parallel resonators to keep the *h*_Cu_/*λ* at the proper range simultaneously. Figure 4a presents the schematic of the proposed thickness-modulated configuration. In contrast to the conventional design, the electrode thicknesses of the series and parallel resonators, referred to as *h*_1_ and *h*_2_, were no longer designed to be the same. A thin Cu electrode *h*_1_ was set for the series resonators, while a thick Cu electrode *h*_2_ was adopted for the parallel resonators. Therefore, the *h*_1_/*λ*_s_ of the series resonators and the *h*_2_/*λ*_p_ of the parallel resonators could be modulated separately so that their normalized thicknesses could be kept in the optical range simultaneously. Specifically, two different designs were proposed and are presented in Figure 4b,c, respectively. The first one controlled the *h*_1_/*λ*_s_ of the series resonators and the *h*_2_/*λ*_p_ of the parallel resonators at about 0.1, where the coupling strength of the Rayleigh mode was the weakest. In the second design, the *h*_2_/*λ*_p_ of the parallel resonator was still 10%, while the *h*_1_/*λ*_s_ of the series resonator could be set more than 10% so that the response of the Rayleigh wave moved outside of the passband and no longer affected the filter passband. Using the two proposed configurations, the spurious modes in the wideband SAW filters were expected to be suppressed entirely. Moreover, it should be pointed out that the proposed configuration could be easily realized in two photolithography steps and would not incur too much complexity or cost for device fabrication.

### 3.3. Design and Fabrication of SAW Filter Using First IDT Thickness Modulated Configuration

**Figure 5:**
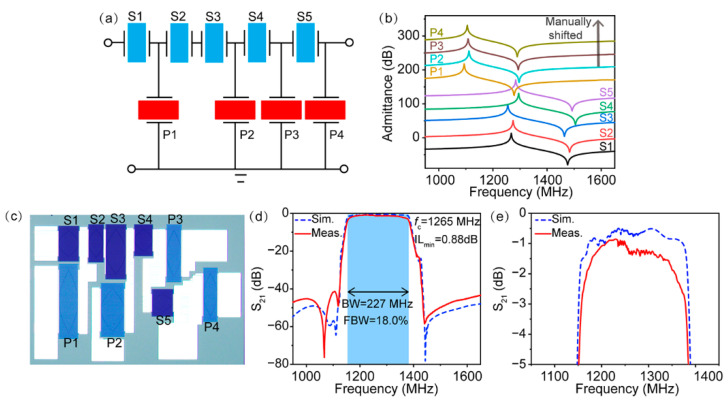
(**a**) The filter topology; (**b**) simulated admittance curves of series and shunt resonators. The curves were manually shifted by 40 dB for better comparison; (**c**) optical microscope of the fabricated filter; (**d**) simulated and measured frequency responses of the filter; (**e**) zoomed-in simulated and measured frequency responses of the filter.

We fabricated wideband SAW filters on the 15°YX-LiNbO_3_ substrate according to the two IDT thickness-modulated designs proposed above to further verify our idea. Figure 5a shows the topography of ladder-type SAW filters consisting of five series resonators and four parallel resonators, abbreviated as S1–S5 and P1–P4, respectively. The design parameters of the SAW filter are provided in Table 1. It should be mentioned that since the packaging structure has an effect on the electrical performance of the SAW filter, the electromagnetic parasitic effects (the filter layout, the interconnections, the multilayer laminate substrate, the die sits on, the package et al.) were considered concurrently during the design process to correctly simulate the performance of the SAW filters in our work. The series and parallel resonators adopted different IDT electrode thicknesses to keep their normalized electrode thickness *h*_Cu_/*λ* at around 10% synchronously, with the Rayleigh modes possessing the weakest resonance according to FEM simulation. Figure 5b illustrates the simulated admittance curves of the nine corresponding resonators in the filter. Note that all of the series and parallel resonators showed a clean spectrum revealing the thorough mitigation of Rayleigh mode. 

The SAW filter was fabricated using two-step photolithography, and a standard surface-mounted device that was 3 × 3 mm^2^ in size was packaged. Figure 5c shows a typical optical image of the fabricated filter. Obviously, the contrast between the series and parallel resonators under the optical microscope was different due to the differences in thickness of the Cu electrode. The measured and simulated frequency responses of the SAW filter are shown in Figure 5d,e. It is worth noting that the measured performance coincided well with the simulated frequency response, suggesting the accuracy of our design. The measured filter showed a center frequency (*f*_c_) of 1265 MHz, a high 3 dB FBW of 18.0%, a low IL_min_ of 0.88 dB, and a high out-of-band rejection of nearly 40 dB. Additionally, since the undesired Rayleigh modes in the series and parallel resonators were able to be eliminated well when using the proper IDT electrode thickness, a flat in-band response was achieved, fully satisfying the demands of commercial applications.

### 3.4. Design and Fabrication of SAW Filter Using Second IDT Thickness Modulated Configuration

**Figure 6:**
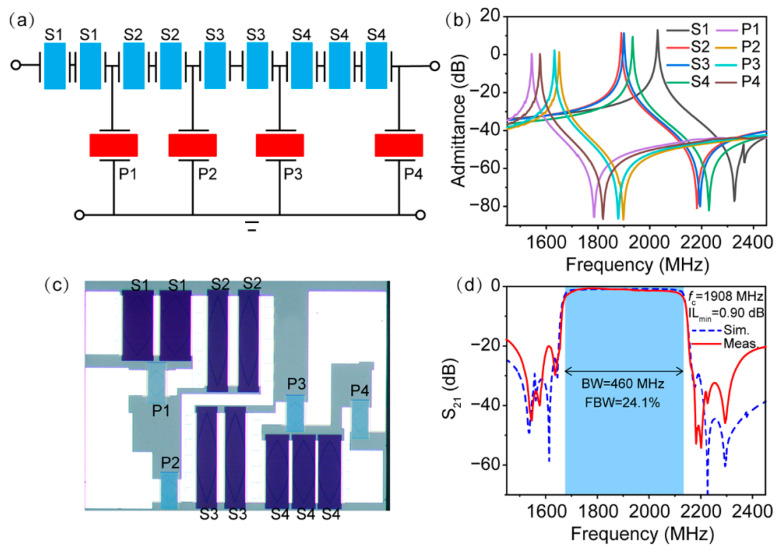
(**a**) The filter topology; (**b**) simulated admittance curves of series and shunt resonators; (**c**) optical microscope of the fabricated filter; (**d**) simulated and measured frequency responses of the filter.

We next investigated SAW filters under the second design scheme. The topology of the filter is shown in Figure 6a and consists of four series resonators (abbreviated as S1–S4) and four parallel resonators (abbreviated as P1–P4). Double or triple series resonators were adopted to avoid extremely small resonators or a small aperture length. Moreover, to design a SAW filter with high accuracy, the package parasitics on the performance of SAW filters were also taken into account. The key parameters of the filter are summarized in Table 2. With the exception of the the *h*_Cu_/*λ* of the resonator S1 exceeding 10%, the thicknesses of all of the other resonators were designed to be around 10% to guarantee the complete removal of spurious responses according to the aforementioned principle. Figure 6b shows the simulated admittance curves of all of the corresponding resonators in the filter. It is clearly seen that with the exception of series resonator S1, there was no spurious Rayleigh mode in the other resonators. From Figure 1, we can determine that the *h*_Cu_/*λ* thickness of over 10% used in resonator S1 made the Rayleigh mode move to the right of the anti-resonant frequency of the SH mode. Consequently, the response of the Rayleigh wave occurred outside of the passband and ensured a spurious-free SH resonance in the filter passband. 

Following the design parameters above, SAW filters were prepared and placed in chip-scale packages that were 1.4 × 1.1 mm^2^ in size. The optical image of the fabricated SAW filter is displayed in Figure 6c. Similar to the above results, the thickness-induced contrast of series and shunt resonators can also be seen in the optical image. The simulated and measured frequency responses of the filter are shown in Figure 6d. Apparently, consistent with the design in Figure 4c, the simulated filter featured a flat passband with a low insertion loss and a very large FBW of over 24%. Indeed, the spurious mode originating from the Rayleigh mode of the resonator S1 appeared outside of the filter passband, but this can be neglected since the small ripple outside of the filter passband does not affect the in-band performance of the SAW filter. The measured response of the filter confirms the simulation results. The measured *f*_c_ of the fabricated filter was located at 1908 MHz and showed a fairly wide BW of 460 MHz, corresponding to a 3-dB FBW of 24.1%, whereas the IL_min_ was 0.90 dB, confirming the wideband and low-loss performance of the filter. Furthermore, no ripples were observed from spurious Rayleigh modes in the filter passband, and the in-band fluctuations were controlled below 1 dB, indicating effective spurious mode mitigation.

The experimental results concluded that the problem of the existence of spurious Rayleigh modes in the 15°YX-LiNbO_3_ substrate that has acted as an obstacle for SAW filters can be perfectly solved by employing thickness-modulated IDT structures. Moreover, it should also be pointed out that YX-LiNbO_3_ are commercial substrates that are commonly used in the current SAW industry, and the device was fabricated using a standard optical lithography process. Compared to previous work based on LN/SiO_2_/Si platforms [17], the FBW and passband flatness are simultaneously improved, while the preparation costs are greatly reduced. Hence, the low-cost mass production of spurious-free ultra-wideband SAW filters appears to be quite feasible when using our proposed approach. It is believed that the modulating the IDT thickness the resonator via the method proposed in this work can also be applied to manage spurious modes in other types of acoustic devices.

## 4. Conclusions

In summary, a double thickness-modulated IDT structure in series and shunt resonators was proposed to develop ultra-wideband and spurious-free SAW filters. With the help of FEM simulation, the coupling between the SH and Rayleigh modes can be modulated by the electrode thickness. Using an elaborate IDT thickness design, both the series and shunt resonators comprising the filters were kept in the optimized metal thickness range, completely suppressing unwanted spurious Rayleigh modes or moving them out of the passband. Based on the proposed method, an ultra-wideband SAW filter with a large 3-dB FWB in the 18.0% and 24.1% ranges exhibiting spurious-free and low-loss responses was successfully designed and implemented. The superb performance of these devices showed that this method could provide a promising and compelling solution for the realization of ultra-wideband spurious-free SAW filters and possess the prospect of practical implementations in 5G RF front-end.

## Figures and Tables

**Table 1:** The design parameters of the SAW filter using the first IDT thickness-modulated configuration.

Resonator	*h*_Cu_ (nm)	*λ* (μm)	*h*_Cu_/λ	Metallization Ratio	Number of IDT Electrodes	Number of Reflector
S1	220	2.26	9.73%	0.5	112	20
S2		2.24	9.82%	0.5	123	20
S3		2.30	9.56%	0.5	191	20
S4		2.19	10.05%	0.5	99	20
S5		2.22	9.91%	0.5	79	20
P1	255	2.61	9.77%	0.5	237	20
P2		2.55	10.00%	0.5	163	20
P3		2.56	9.96%	0.5	177	20
P4		2.57	9.92%	0.5	163	20

**Table 2:** The design parameters of the SAW filter using the second IDT thickness-modulated configuration.

Resonator	*h*_Cu_ (nm)	*λ* (μm)	*h*_Cu_/λ	Metallization Ratio	Number of IDT Electrodes	Number of Reflector
S1	140	1.28	10.93%	0.5	237	20
S2		1.46	9.56%	0.5	307	20
S3		1.45	9.66%	0.5	307	20
S4		1.41	9.92%	0.5	223	20
P1	165	1.83	9.02%	0.5	69	20
P2		1.65	10.00%	0.5	69	20
P3		1.68	9.82%	0.5	69	20
P4		1.78	9.27%	0.5	69	20

## Data Availability

The data presented in this study are available upon request from the corresponding author.
